# Supplementary material for: 3D sedimentary architecture showing the inception of an Ice Age
Source: Nat Commun. 2020 Jun 12;11:2975. doi: 10.1038/s41467-020-16776-7 (PMC7293307; doi:10.1038/s41467-020-16776-7)
Supplement: Supplementary file 1 — Supplemmentry Information [file 41467_2020_16776_MOESM1_ESM.pdf]

Supplementary Information

3D sedimentary architecture showing the inception of an Ice Age

Løseth *et al*.

Supplementary Figures

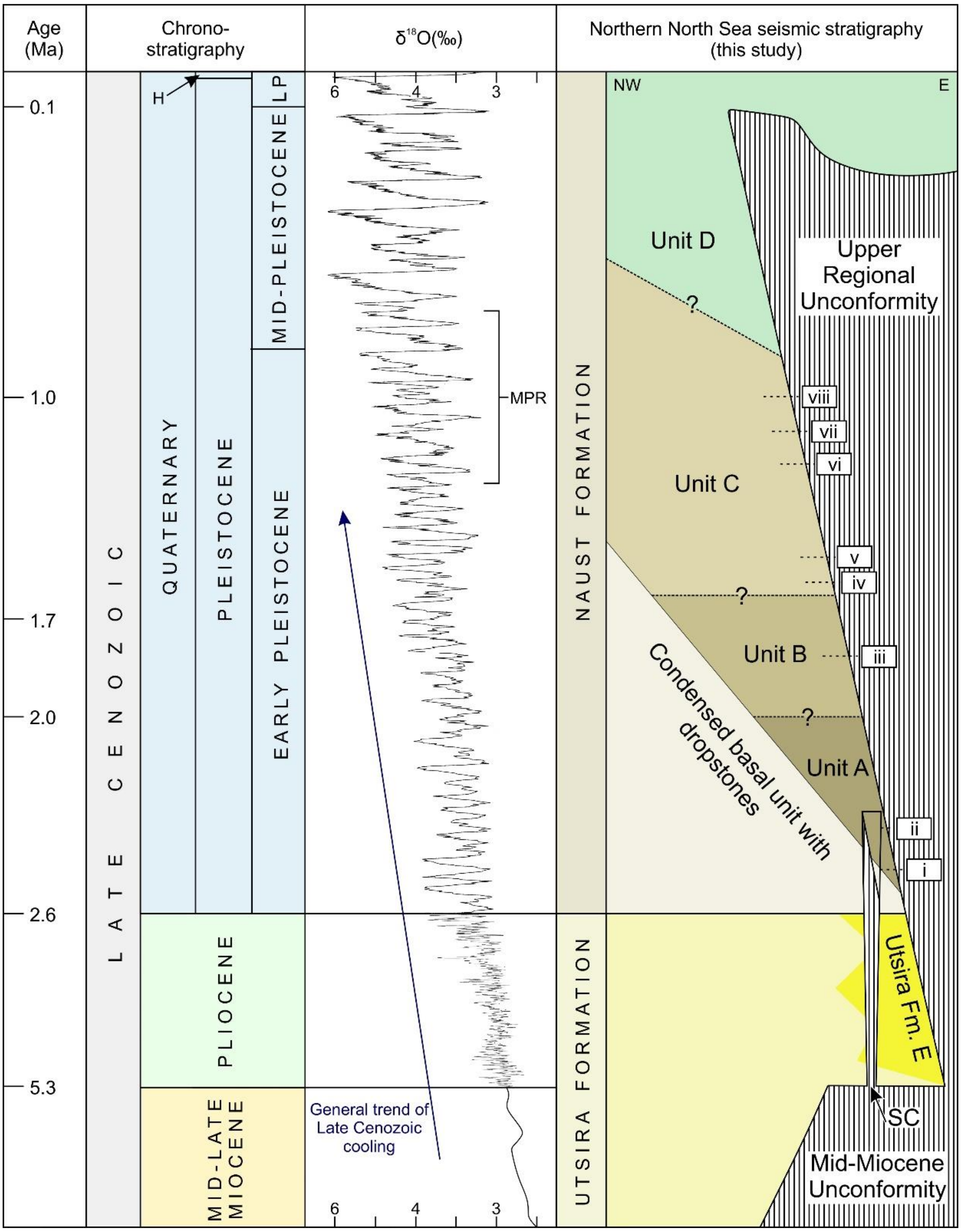

Supplementary Figure 1. Table showing how our tentative ages for Units A to D of the Naust Formation in the northern North Sea correspond with the d^{18}O record of global sea level change since the Mid-Late Miocene. Note that the scale of the vertical axis changes across the Quaternary, Pliocene and Miocene. See Methods for discussion of the age of the Utsira Formation and Units A to D of the Naust Formation. The benthic δ^{18}O stack for the Quaternary and Pliocene is from Lisiecki and Raymo[1], and the smoothed benthic δ^{18}O stack for the Mid-Late Miocene is from Zachos *et al.*[2]. H = Holocene; LP = Late Pleistocene; MPR = Mid-Pleistocene Revolution; SC = Sunnfjord channel.

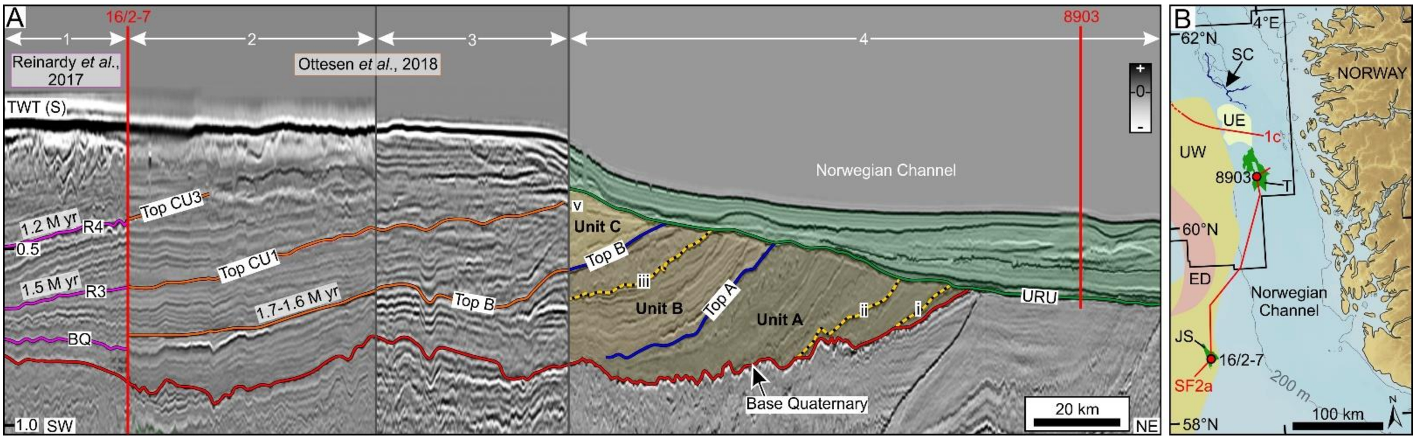

Supplementary Figure 2. Seismic tie of dated horizons from the south into the northern North Sea study area. **(A)** Seismic section from the Johan-Sverdrup Field and well 16/2-7 to the Troll Field and core 8903. The seismic section is composed of the following seismic lines: 1 = ST12M02 inline 4812 (south); 2 = PGS MC3D-NVGSVGM2013 crossline 12820; 3 = NSR08-42359; 4 = CGG18M01 random composite line (north). The interpreted horizons from Reinardy *et al.*[3] and Ottesen *et al.*[4] are shown in pink and orange, respectively. Within CGG18M01 (seismic line 4), the colours are the same as in Fig. 1c. In addition to the tied ages shown in this figure, we tentatively suggest an age of 2.2–2.0 M yr for the top of Unit A. See Methods for further discussion of the likely ages of these sediments. BQ = Base Quaternary of Reinardy *et al.*[3] (note that this is different from the Base Quaternary used in Ottesen *et al.*[4] and this study, which is shown in red). CU = Clinoform Unit; TWT = two-way travel time; URU = Upper Regional Unconformity. **(B)** Map showing the location of the seismic section presented in (A). The solid black line shows the extent of 3D seismic cube CGG19M01. Green filled areas are Johan-Sverdrup (JS) and Troll (T) fields. The distribution of the Utsira Formation west (UW) is from Eidvin *et al.*,[5]. ED = Early Quaternary deltaic unit; SC = Sunnfjord channel; SF = Supplementary Figure; UE = Utsira Formation east.

Supplementary References

1. Lisiecki, L. E. & Raymo, M. E. A Pliocene-Pleistocene stack of 57 globally distributed benthic δ18O records. *Paleoceanogr. Paleocl.* **20**, PA1003 (2005).
2. Zachos, J. C., Dickens, G. R. & Zeebe, R. E. An early Cenozoic perspective on greenhouse warming and carbon-cycle dynamics. *Nature* **451**, 279–283 (2008).
3. Reinardy, B. T. I., Sejrup, H. P., Hjelstuen, B. O., King, E. & Augedal, H. A Quaternary aminostratigraphy constraining chronology of depositional environments in the North Sea Basin. *Mar. Geol.* **402**, 139–152 (2017).
4. Ottesen, D., Batchelor, C. L., Dowdeswell, J. A. & Løseth, H. Morphology and pattern of Quaternary sedimentation in the North Sea Basin (52–62ºN). *Mar. Petrol. Geol.* **98**, 836–859 (2018).
5. Eidvin, T., Riis, F. & Rasmussen, E. S. Oligocene to Lower Pliocene deposits of the Norwegian continental shelf, Norwegian Sea, Svalbard, Denmark and their relation to the uplift of Fennoscandia: A synthesis. *Mar. Petrol. Geol.* **56**, 184–221 (2014).
